# Reductive Transformation of Fe(III) (oxyhydr)Oxides by Mesophilic Homoacetogens in the Genus *Sporomusa*

**DOI:** 10.3389/fmicb.2021.600808

**Published:** 2021-02-01

**Authors:** Kensuke Igarashi, Souichiro Kato

**Affiliations:** ^1^Bioproduction Research Institute, National Institute of Advanced Industrial Science and Technology (AIST), Sapporo, Japan; ^2^Division of Applied Bioscience, Graduate School of Agriculture, Hokkaido University, Sapporo, Japan

**Keywords:** homoacetogenic bacteria, iron reduction, mineral transformation, extracellular electron transfer, element cycle

## Abstract

Microbial reduction of iron contributes to the dissolution and transformation of iron-containing minerals in nature. Diverse groups of homoacetogenic bacteria (homoacetogens) have been reported to reduce insoluble Fe(III) oxides, such as hydrous ferric oxide (HFO), an Fe(III) mineral commonly found in soils and sediments. Several members of genus *Sporomusa* reportedly oxidize Fe(0), indicating the presence of an extracellular electron-uptake mechanism. However, the ability of the genus to reduce insoluble Fe(III) oxides is limited, and the underlying reduction mechanism remains to be elucidated. In this study, the HFO reduction ability of three *Sporomusa* spp. (*Sporomusa* sp. strain GT1, *Sporomusa sphaeroides*, and *Sporomusa ovata*) and a homoacetogen of a different genus (*Acetobacterium woodii*) were assayed under organotrophic (ethanol) and lithotrophic (H_2_ + CO_2_) conditions without a chelator or reducing reagent. All tested homoacetogens showed acetogenic growth and concomitant reduction of HFO under both organotrophic and lithotrophic conditions. Analysis of the growth stoichiometry showed that Fe(III) reduction does not support direct energy conservation, thereby indicating that Fe(III) reduction is a side reaction of acetogenesis to dissipate the excess reducing power. HFO was reduced to a soluble Fe(II) form by microbial activity. In addition, we observed that strain GT1, *S. sphaeroides*, and *S. ovata* reduced crystalline Fe(III) oxides, and HFO was reductively transformed into magnetite (Fe_3_O_4_) under phosphate-limiting conditions. Separation of HFO by a dialysis membrane still permitted Fe(II) production, although the reduction rate was decreased, suggesting that Fe(III) reduction is at least partially mediated by soluble redox compound(s) secreted from the cells. Finally, culture experiments and comparative genomic analysis suggested that electron transfer by flavins and multiheme *c*-type cytochrome were not directly correlated with Fe(III) reduction activity. This study reveals the capability of *Sporomusa* spp. in the reductive transformation of iron mineral and indicates the potential involvement of these organisms in iron and other mineral cycles in nature.

## Introduction

Microbial iron reduction greatly influences the dissolution, accumulation, and transformation of iron-bearing minerals in natural environments. In addition, such reactions induce mineralization of other elements, thus affecting the global cycles of various elements (Weber et al., 2006). Insoluble Fe(III) oxides, such as hydrous ferric oxide (HFO) [or a poorly crystalline Fe(III) oxyhydroxide], are the dominant phase of Fe(III) in the neutral pH range and used by numerous microbes as an electron sink for their metabolism, particularly in anaerobic environments (Thamdrup, 2000; Lovley et al., 2004; Richter et al., 2012; Lovley, 2013).

Phylogenetically diverse bacteria have been shown to reduce soluble Fe(III) forms and insoluble Fe(III) (i.e., iron oxides) in indirect and/or direct manners (Hammann and Ottow, 1974, 1976; Hyun et al., 1999; Benner et al., 2002; Lovley, 2013). Such bacteria are known as iron-reducing bacteria (IRBs). Many studies have focused on several model IRBs, such as Gram-negative proteobacteria, *Geobacter* (Lovley et al., 2004), and *Shewanella* spp. (Hyun et al., 1999), and Gram-positive Firmicutes bacteria belonging to genus *Thermincola* (Wrighton et al., 2011) and *Listeria* (Light et al., 2018). Molecular biological studies of these IRBs have revealed that the ability to directly reduce Fe(III) is involved in the extracellular electron transfer (EET) system depending on membrane-binding multiheme *c*-type cytochromes (MHCs), which transfer electrons sequentially from the quinone pool in the cytoplasmic membrane, across the periplasm, to the outer cell surface (Weber et al., 2006; Shi et al., 2007; Inoue et al., 2010; Carlson et al., 2012). Recent studies reported that diverse Gram-positive bacteria can employ a flavin-based extracellular electron transfer mechanism (Light et al., 2018). In this mechanism, electrons from NADH in the cytoplasm are transferred to extracellular flavin, after which reduced flavin transfers the electrons to other electron acceptors outside the cell (Light et al., 2018). However, whether other non-model IRBs employ the same or similar molecular mechanism for iron reduction remains unclear.

Homoacetogenic bacteria (homoacetogens) are a group of bacteria that can produce acetate from C1 compounds *via* the reductive acetyl-CoA pathway and are found mostly in the phylum Firmicutes (Ljungdahl, 1969; Wood et al., 1986; Ragsdale and Pierce, 2008). They dominate various environments, particularly anaerobic water, soil, and sediments, where they catalyze the degradation of organics by fermentative metabolism. In addition, they play an essential role in primary production by homoacetogenic metabolism in anaerobic environments. Various homoacetogens can reduce soluble and insoluble Fe(III) (Dobbin et al., 1999; Sass et al., 2004; Slepova et al., 2009; Yoneda et al., 2012; Dalla Vecchia et al., 2014; Yang et al., 2016). Most studies have focused on thermophilic homoacetogens, such as members of *Thermolithobacter and Carboxydothermus* (Slobodkin et al., 1997; Sokolova et al., 2007; Slepova et al., 2009; Gavrilov et al., 2012; Yoneda et al., 2012) and mesophilic variants, such as the members of *Desulfotomaculum* (Dalla Vecchia et al., 2014; Yang et al., 2016); however, few studies have reported insoluble Fe(III) reduction by mesophilic homoacetogens affiliated with class *Negativicutes* (i. g., *Desulfosporomusa polytropa*) (Sass et al., 2004). A notable characteristic of the class is that cells harbor an outer membrane over the murein layer, forming a multilayered Gram-negative cell wall (Möller et al., 1984; Marchandin et al., 2010; Campbell et al., 2014), suggesting that the outer cell-surface components would differ from those of other classes within the Firmicutes.

Bacteria from the genus *Sporomusa* have been described in 1984 (Möller et al., 1984) and their phylogenetic position within the class *Negativicutes* was assigned decades later (Marchandin et al., 2010). Bacteria in the genus distribute broadly in anoxic environments and can use versatile substrates (Breznak, 2006). However, no study has been reported for insoluble Fe(III) reduction by the genus. *Sporomusa* spp. have attracted considerable attention because of their capability of electron uptake from an electrode for CO_2_ reduction in the microbial electrochemical system (Nevin et al., 2010; Aryal et al., 2017). Our previous study revealed that the homoacetogens *Sporomusa* sp. GT1 and *Sporomusa sphaeroides* (Möller et al., 1984) induce extensive oxidation of metallic iron [Fe(0)] under homoacetogenic growth conditions (Kato et al., 2015). This finding indicates the presence of an electron transfer mechanism for electron uptake. Our previous results also implied that such electron transfer ability may reflect a strong electrochemical association between naturally occurring (semi)conductive iron minerals (Kato et al., 2015). Thus, we expected that *Sporomusa* spp. can also induce insoluble iron reduction.

In this study, we investigated the ability of strain GT1 and *S. sphaeroides* to reduce HFO in the absence of a soluble electron shuttle under organotrophic and lithotrophic culture conditions. For comparison, we also assayed Fe(III) reduction by two homoacetogenic strains that do not show Fe(0) oxidation activity [i.e., *Sporomusa ovata* (Möller et al., 1984) and *Acetobacterium woodii* (Balch et al., 1977)] (Kato et al., 2015). The reduction products were investigated to evaluate the impact of homoacetogens on iron dissolution and transformation in nature. Additionally, we evaluated the ability of the four homoacetogens to reduce soluble and crystalline Fe(III) oxides. Finally, culture experiments were performed to test the involvement of a soluble electron shuttle/chelator, and comparative genomics analysis was conducted to obtain insight into the molecular mechanism underlying HFO reduction by the homoacetogens.

## Materials and Methods

### Microorganisms and Cultivations

*Sporomusa sphaeroides* DSM2875, *S. ovata* DSM2662, and *A. woodii* DSM1030 were obtained from the Deutsche Sammlung von Mikroorganismen und Zellkukturen GmbH (Braunschweig, Germany). *Sporomusa* sp. strain GT1 was isolated in our previous study (Kato et al., 2015) and maintained using routine procedures. All anaerobic cultivations in this study except for the experiments using the dialysis membrane (see below) were conducted in a sealed glass bottle (68 mL in capacity), as described previously (Kato et al., 2015), filled with 20 mL of freshwater medium at 30°C in the dark without agitation. The medium contained the following reagents per liter of distilled water: 0.3 g of KH_2_PO_4_; 1 g of NH_4_Cl; 0.1 g of MgCl_2_⋅7H_2_O; 0.08 g of CaCl_2_⋅7H_2_O; 0.6 g of NaCl; 2 g of KHCO_3_; 9.52 g of 4-(2-hydroxyethyl)-1-piperazine ethane sulfonic acid (HEPES); 0.1 g yeast extract; and 10 mL each of trace metal solution and vitamin solution (Sekiguchi et al., 2000). The pH of the medium was adjusted to 7.4 by adding 6 N KOH solution. When culturing the cells under phosphate-limiting conditions, the concentration of KH_2_PO_4_ was decreased to 0.05 g/L. Unless otherwise mentioned, 100 kPa of N_2_ + CO_2_ (80:20) gas phase was used. All culture media were vigorously bubbled with the gas mixture (N_2_ + CO_2_) for at least 20 min to remove dissolved oxygen and then autoclaved at 121°C for 20 min. Yeast extract (0.1 g/L) was required for stable growth of the homoacetogens tested in this study. Organotrophic growth using yeast extract was assessed and found to reach a maximum of 6.0 × 10^6^ cells/mL, which is less than *ca.* 2% of full growth on ethanol and H_2_ + CO_2_ (see below). Riboflavin and flavin adenine dinucleotide (FAD) were added to the medium at 5 μM, as necessary.

Hydrous ferric oxide was prepared as described previously (Lovley and Phillips, 1988) and suspended in Milli-Q water (0.3 mol/L) under an N_2_ atmosphere. HFO-amended medium was prepared by adding an HFO suspension to an initial concentration of 9 mmol/L. Goethite [α-FeO(OH)] and hematite (α-Fe_2_O_3_) as crystalline Fe(III) oxides were tested instead of HFO. Goethite was synthesized as described previously (Lovley and Phillips, 1986), and hematite was purchased from Kojundo Chemical Laboratories (Saitama, Japan) (Igarashi et al., 2016). The suspensions of these Fe(III) oxides were prepared under the same conditions described in this section and added to the medium to ensure that contained the same number of Fe(III) atoms as HFO-amended medium. For comparison to insoluble Fe(III) (hydro)oxides, Fe(III)-nitrilotriacetic acid (NTA) in its soluble Fe(III) form was added to the medium; however, the initial concentration was set to 2 mM, because inhibitory effects on growth were observed at concentrations ≥ 3 mM.

Cultures of four homoacetogens were maintained by routinely transferring exponentially growing cultures (*ca*. 5–10% volume) to fresh medium supplemented with ethanol (20 mM). Precultures were conducted at least twice in the presence of each substrate and Fe(III). Exponentially growing cultures were used for Fe(III) reduction experiments. Organotrophic cultures for HFO reduction were started on ethanol (20 mM). For lithotrophic culture, cultures were started with 200 kPa of H_2_ + CO_2_ (80:20) gas phase without ethanol, providing approximately 3.2 mmol H_2_ per bottle. The initial cell density of each homoacetogen was 1.0 × 10^6^ cells/mL. During cultivation, the cultures were periodically sampled with 2- or 4-day intervals for cell density and chemical analyses. All culture experiments were conducted in triplicate. Tukey’s honest significance difference test was conducted for statistical analysis.

### Incubation With Spent Medium

Stationary phase cultures of each acetogen on ethanol were filtered through a 0.2-μm pore filter in an anaerobic chamber (Coy Laboratory Products; Grass Lake, MI, United States) under a gas mixture of N_2_ + H_2_ + CO_2_ (80:5:15). The filtrate (18 mL) and HFO (9 mmol/L as final concentration) were placed in a fresh culture bottle, supplemented with ethanol (20 mM), and incubated under the same conditions described above.

### Separation of HFO by a Dialysis Membrane

Cultivation with physically separated HFO from cells was conducted using a dialysis device (Spectra/Por^®^ Float-A-Lyzer^®^ G2; MWCO 20 KDa; REPLIGEN; Waltham, MA, United States). The medium volume was increased by sixfold (120 mL) in the DURAN^®^ laboratory bottle (290 mL in capacity) to accommodate the dialysis device inside the culture container. Preparation of culture using the dialysis device was conducted inside the anaerobic chamber. The dialysis device was pre-treated according to the manufacturer’s instructions. The device was sterilized by rinsing 70% ethanol and stored in sterilized Milli-Q water until use. The suspension of HFO was loaded into the device to give the same Fe(III) input per medium volume as used in the culture experiment described above (see section “Microorganisms and cultivations”). The device was sealed and floated in the medium. Each of homoacetogens was inoculated into the medium to obtain the same cell density as the normal culture condition using 20 mL medium (1.0 × 10^6^ cells/mL). After sealing with a rubbers stopper and plastic screw cap, the bottle was removed from the anaerobic chamber and the headspace gas was replaced with N_2_ + CO_2_ (80:20). Cultivation was conducted at 30°C for 14 days. The potential inhibitory effects of the device on cell growth were tested by conducting a control culture using an empty dialysis device and medium amended with HFO and ethanol. After cultivation, the integrity of the dialysis tube was confirmed by checking the potential microbial contamination inside the device by fluorescent microscopy using a LIVE/DEAD *BacLight*^TM^ Bacterial Viability Kit (LIVE/DEAD; Molecular Probes, Eugene, OR, United States) as described previously (Igarashi and Kuwabara, 2014). The Fe(II) concentration in the culture medium (outside the device) was quantified as described below.

### Cell Density Determination

The density of planktonic cells was quantified by direct cell counting as described previously (Igarashi et al., 2020). Briefly, during cultivation, 100 μL of culture was periodically sampled with a disposable syringe. The sample was mixed with a LIVE/DEAD *BacLight*^TM^ Bacterial Viability Kit and then cells were directly counted using a bacterial counting chamber and a BX50 optical microscope (Olympus, Tokyo, Japan).

### Chemical Analysis

The acetate and ethanol concentrations in the liquid phase were determined by using a high-performance liquid chromatography system (D-2000 LaChrom Elite HPLC system; Hitachi, Tokyo, Japan) equipped with an Aminex^®^ HPX-87H column (300 mm, 7.8 mm I.D.; Bio-Rad Laboratories, Hercules, CA, United States), ultraviolet detector at 240 nm (L-2400, Hitachi), and refractive index (L-2490, Hitachi) as described previously (Kato et al., 2015). The liquid sample (200 μL) was periodically collected using a disposable syringe. The filtered sample (10 μL) was directly injected into the HPLC circuit and subjected to chromatography.

Fe(II) produced during cultivation was quantified as described previously (Igarashi and Kuwabara, 2014). To avoid possible oxidation of Fe(II), all procedures except for spectrometric analysis were performed inside the anaerobic chamber. Briefly, the culture bottle was vigorously shaken, and the suspended culture containing insoluble components (100 μL) was collected with a disposable syringe. Twenty microliters of the sample were reacted with 80 μL of 0.625 N HCl to extract Fe(II). Twenty microliters of this extract solution were mixed with 1 mL of 0.1% (w/v) ferrozine in 50 mM HEPES–NaOH (pH 7.0) (Stookey, 1970). After standing at room temperature for 15 min, the mixed sample was centrifuged at 5,000 × *g* for 1 min. The Fe(II) concentration was determined by measuring *A*_562_ using an ultraviolet-VIS spectrometer (V-660, JASCO, Tokyo, Japan). Fe(III) contained in the culture was also quantified after reaction with hydroxylamine according to previously reported methods (Lovley and Phillips, 1987). The Fe(II or III) present in supernatant and sediment was separately determined according to previously reported methods (Igarashi and Kuwabara, 2014), with slight modification. Briefly, samples of homogenized culture were centrifuged at 9,000 × *g*, for 5 min at room temperature inside the anaerobic chamber, after which the amount of Fe(II or III) in the supernatant was determined. The amount of Fe(II or III) in the sediment was calculated by subtracting the soluble Fe(II or III) from total Fe(II or III).

### Powder X-Ray Diffraction

X-ray diffraction (XRD) specimens of culture sediments were prepared anaerobically as described previously (Igarashi et al., 2016). XRD spectra were obtained with a RINT2000 X-ray diffractometer (Rigaku, Tokyo, Japan) for CuKα_1,2_ radiation scanning at a step interval of 0.02° 2θ and counting time of 2 s with a 2θ range of 20°–60°, operating at an accelerating voltage of 40 kV at 30 mA.

### Genome Sequence of Strain GT1

The genomic DNA of strain GT1 was isolated and purified as reported previously (Kato et al., 2020). Extracted DNA was used to generate Illumina shotgun paired-end (2 × 101 base pair) sequence libraries, which were sequenced on an Illumina HiSeq 2500 platform (Illumina, San Diego, CA, United States), yielding 4.3 Gb raw read data. The obtained reads were quality-trimmed with Trimmomatic v0.38 (Bolger et al., 2014) (SLIDINGWINDOW: 6:30, MINLEN: 78, and other parameters by default) and assembled using SPAdes v3.12.1 (Bankevich et al., 2012) (kmers of 21, 33, 41, 55, and 77 nucleotides and other parameters by default). The assembled contigs were annotated using Prokka v1.13 (Seemann, 2014) with default parameters.

### Comparative Genomic Analysis

The genome sequences of *S. sphaeroides* DSM2875, *S. ovata* DSM2662, and *A. woodii* DSM1030 were retrieved from the National Center for Biotechnology Information (NCBI) database. To identify MHCs, the cytochrome motif was searched manually over the genomes of four homoacetogens and automatically by using the EXPASY website^1^. The common heme-binding domain, CxxCH, was searched as the primary analysis (Yee et al., 2019; Yee and Rotaru, 2020). Protein localization of MHCs with a common heme-binding domain was predicted by TMHMM v2.0 (Krogh et al., 2001) and SignalP v 5.0 (Almagro Armenteros et al., 2019). Further analysis was conducted using, besides CxxCH, other variants of heme-binding domains, CxxxCH, CxxxxCH, CxxCK, and A/FxxCH as query sequences (Li et al., 2020). Then, amino acid sequences that possess at least two heme-binding domains were analyzed using the SUPERFAMILY website^2^ to determine structural classification as redox protein, such as multiheme cytochrome, based on Structural Classification of Proteins (SCOP) 1.75 domain assignment.

The presence of genes involved in Fe(III) reduction pathways was confirmed by BLASTP v2.7.1 analysis against homoacetogens genomes using default parameters. Homologous proteins were selected according to an E-value cut-off < 10^–4^ and a sequence-identity cut-off >30%. All query sequences were retrieved from NCBI or Uniprot databases. Amino acid sequences of representative proteins conserved in MHC-dependent Fe(III) reducing bacteria (*cymA*, *mtrA*, *mtrB*, *mtrC*, *pilA*, *omcS*, *ppcA*, and *omcB*) were used as query sequences (Reguera et al., 2005; Garber et al., 2020). In addition, we analyzed the sequences of representative proteins potentially involved in flavin-based electron transfer in Gram-positive bacteria (*fmnA*, *dmkA*, *fmnB*, *pplA*, *ndh2*, *eetA*, *eetB*, and *dmkB*) in the *Listeria monocytogenes* genome (Light et al., 2018). Sequences of proteins involved in riboflavin biosynthesis (*ribBA*, *ribD*, *ribE*, *ribF*, and *ribH*) in *Anoxybacter fermentans* DY22613 (Li et al., 2020) were also tested.

## Results

### Growth-Dependent Reduction of HFO

**FIGURE 1:**
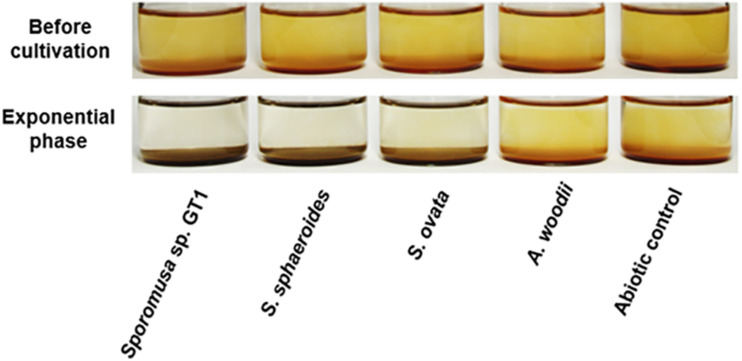
Appearance of homoacetogen cultures in the presence of HFO. Homoacetogens were cultivated on ethanol (20 mM) in the presence of HFO.

**FIGURE 2:**
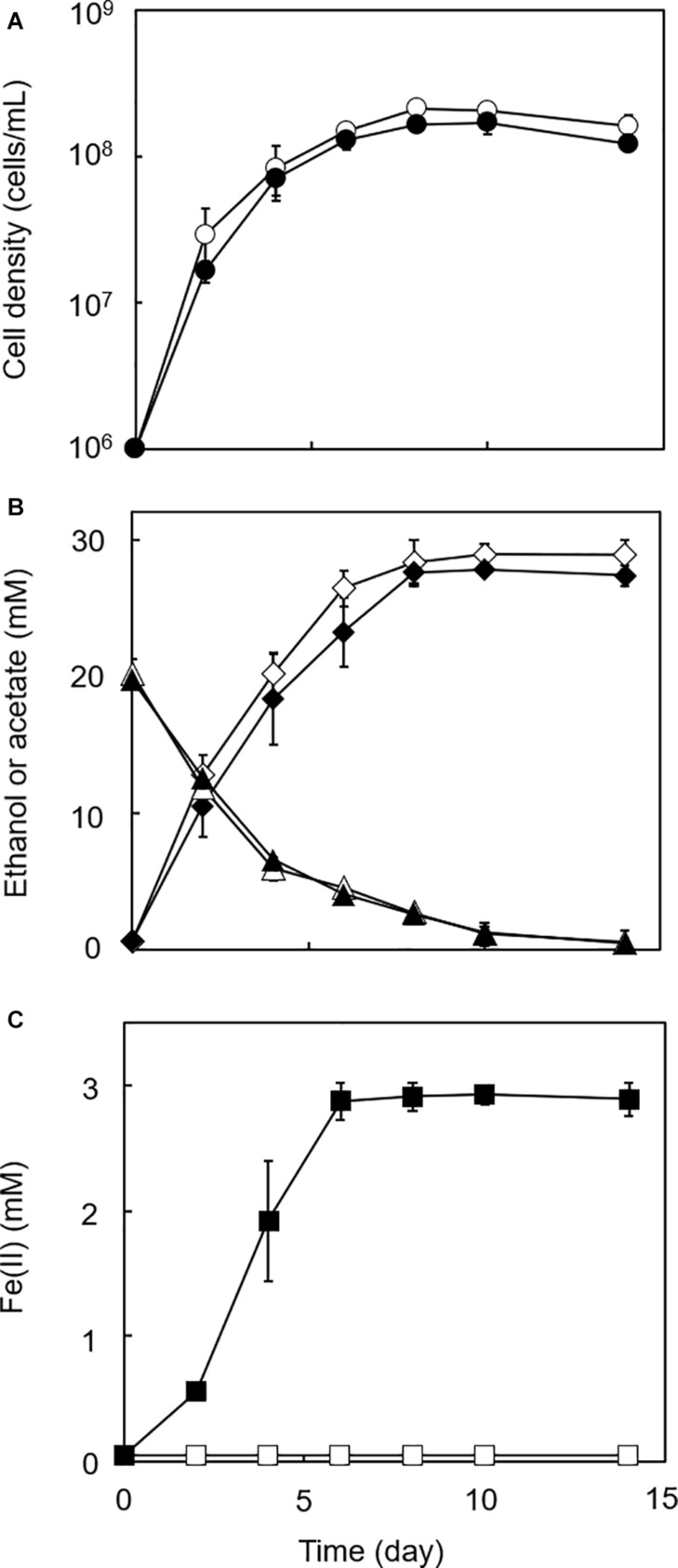
Growth-dependent HFO reduction by strain GT1 under organotrophic conditions. Strain GT1 was cultivated on ethanol (20 mM) in the presence or absence of HFO. **(A)** Cell density, **(B)** concentration of ethanol (triangles) and acetate (diamonds), and **(C)** Fe(II) concentration were periodically determined during cultivation. Closed symbols, cultures with HFO. Open symbols, cultures without HFO. Data are presented as the means of three independent cultures, and error bars represent standard deviations.

Four homoacetogens, *Sporomusa* sp. GT1, *S. sphaeroides*, *S. ovata*, and *A. woodii*, were cultivated in the presence of HFO using ethanol (20 mM) as a substrate. The color of HFO changed from brownish to dark-brownish during cultivation of strain GT1, *S. sphaeroides*, and *S. ovata*. This appearance change indicated mineral transformation by microbial activity (Figure 1). All homoacetogens showed growth and stoichiometric acetogenesis on ethanol (Figure 2 and Supplementary Figure 1). Theoretically, 1 mole of ethanol can be converted to 1.5 moles of acetate according to the reaction schemes shown below (Schuchmann and Müller, 2016).

Ethanol oxidation to acetate:

2 C_2_H_5_OH + 2 H_2_O → 2 CH_3_COOH + 4 H_2_ **(1)**

CO_2_ reduction to acetate:

4 H_2_ + 2 CO_2_ → CH_3_COOH + 2 H_2_O **(2)**

Overall reaction (Eq. 1 + 2):

2 C_2_H_5_OH + 2 CO_2_ → 3 CH_3_COOH **(3)**

**FIGURE 3:**
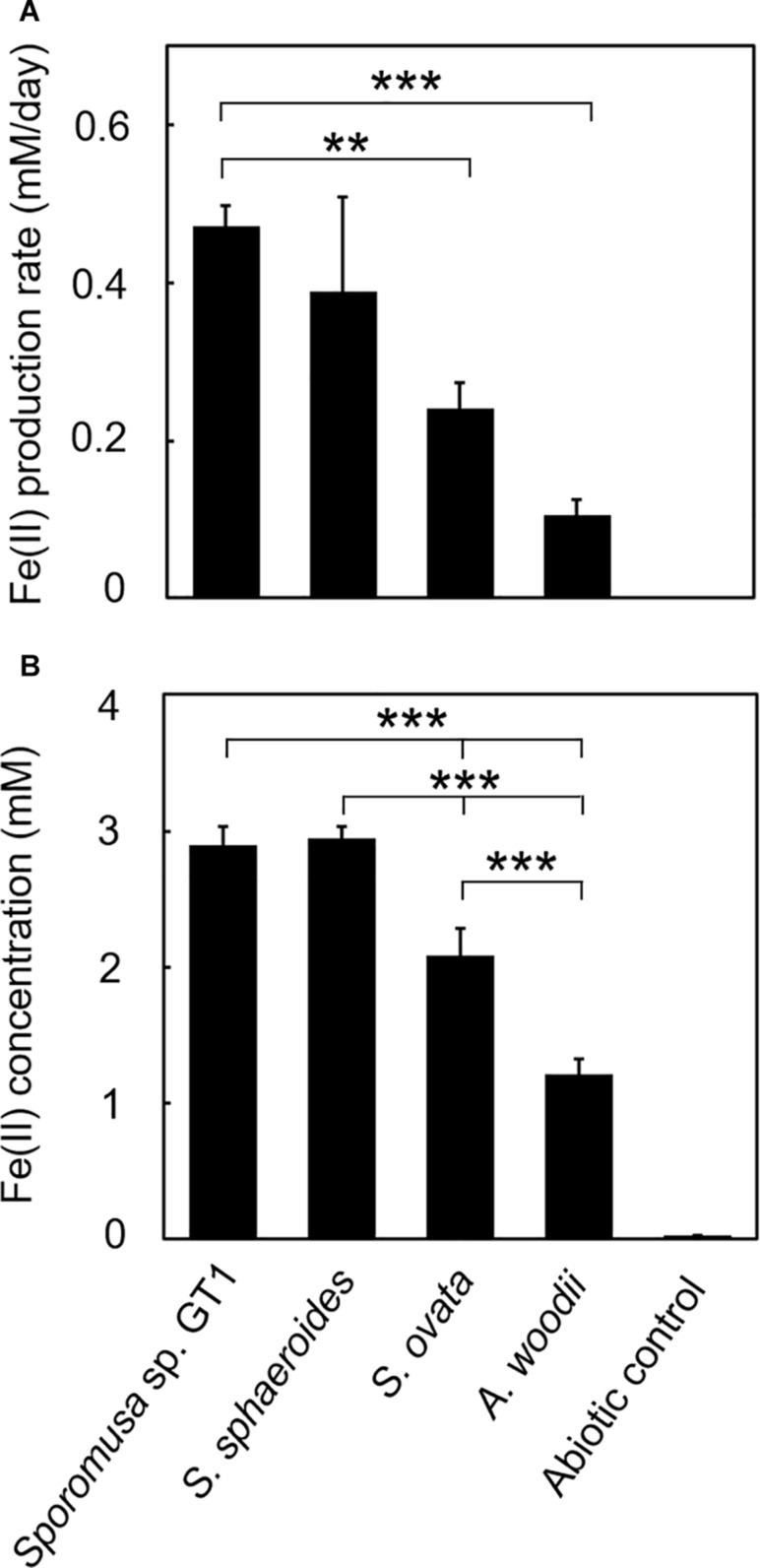
Difference in HFO reduction activity among homoacetogens under organotrophic growth conditions. Homoacetogens were cultivated on ethanol (20 mM) in the presence of HFO. **(A)** Fe(II) production rate in the exponential phase. **(B)** Fe(II) accumulation in the stationary phase. Data are presented as the means of three independent cultures, and error bars represent standard deviations. ***p < 0.001, **p < 0.01, determined by one-way analysis of variance with Tukey’s honest significance difference test.

In the cultures of the four homoacetogens, although the presence of HFO did not notably affect growth or acetogenesis, a growth-dependent increase in Fe(II) was observed, suggesting the microbial reduction of HFO. Growth and acetogenesis did not differ significantly among the four homoacetogens, whereas strain GT1 and *S. sphaeroides* exhibited the highest Fe(II) production rate (Figure 3A) and accumulation (Figure 3B), followed by *S. ovata* and *A. woodii*. The Fe(II) production rates of strain GT1 and *S. sphaeroides* were 0.47 ± 0.03 and 0.39 ± 0.12 mM/day, respectively. The final Fe(II) concentration reached *ca*. 2.9 mM in both species. The observed differences in HFO reduction activities suggest that the ability of extracellular electron transfer differ depending on the species. HFO-amended cultures exhibited 1.5–5.4% lower acetate production compared to control cultures without HFO. For instance, the culture of strain GT1, in which the highest Fe(II) production was observed, showed a decrease in acetate production in HFO-amended culture (31 ± 3.5 μmol) as compared with that in the control culture; thus, *ca*. 248 μmol electron was expected to be diverted to metabolic flow other than acetate production (Eq. 2). However, the observed Fe(II) production was 58 ± 1.4 μmol. This suggests that homoacetogens can reduce HFO by using a limited number (∼24%) of electrons acquired from an electron donor. Under organotrophic growth conditions, 13–33% of Fe(III) added was reduced to Fe(II) during the tested growth period (14 days). Analysis of soluble and sediment-associated iron showed that HFO was reductively dissolved in the liquid phase during cultivation (Supplementary Figure 2).

**FIGURE 4:**
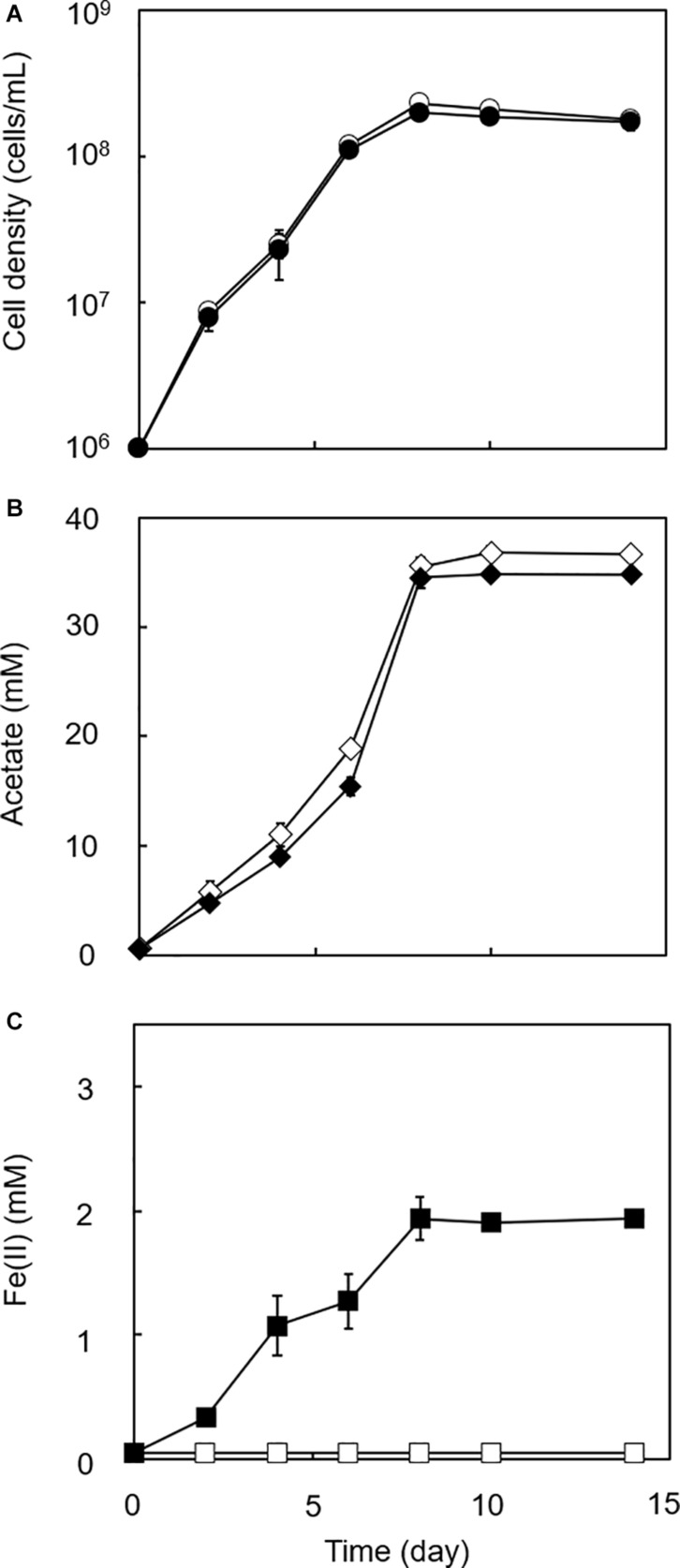
Growth dependent HFO reduction by strain GT1 under lithotrophic condition. Strain GT1 was cultivated on H_2_ + CO_2_ (80:20) in the presence or absence of HFO. **(A)** Cell density, **(B)** concentration of acetate, and **(C)** Fe(II) concentration were periodically determined during cultivation. Closed symbols, cultures with HFO. Open symbols, cultures without HFO. Data are presented as the means of three independent cultures, and error bars represent standard deviations.

**FIGURE 5:**
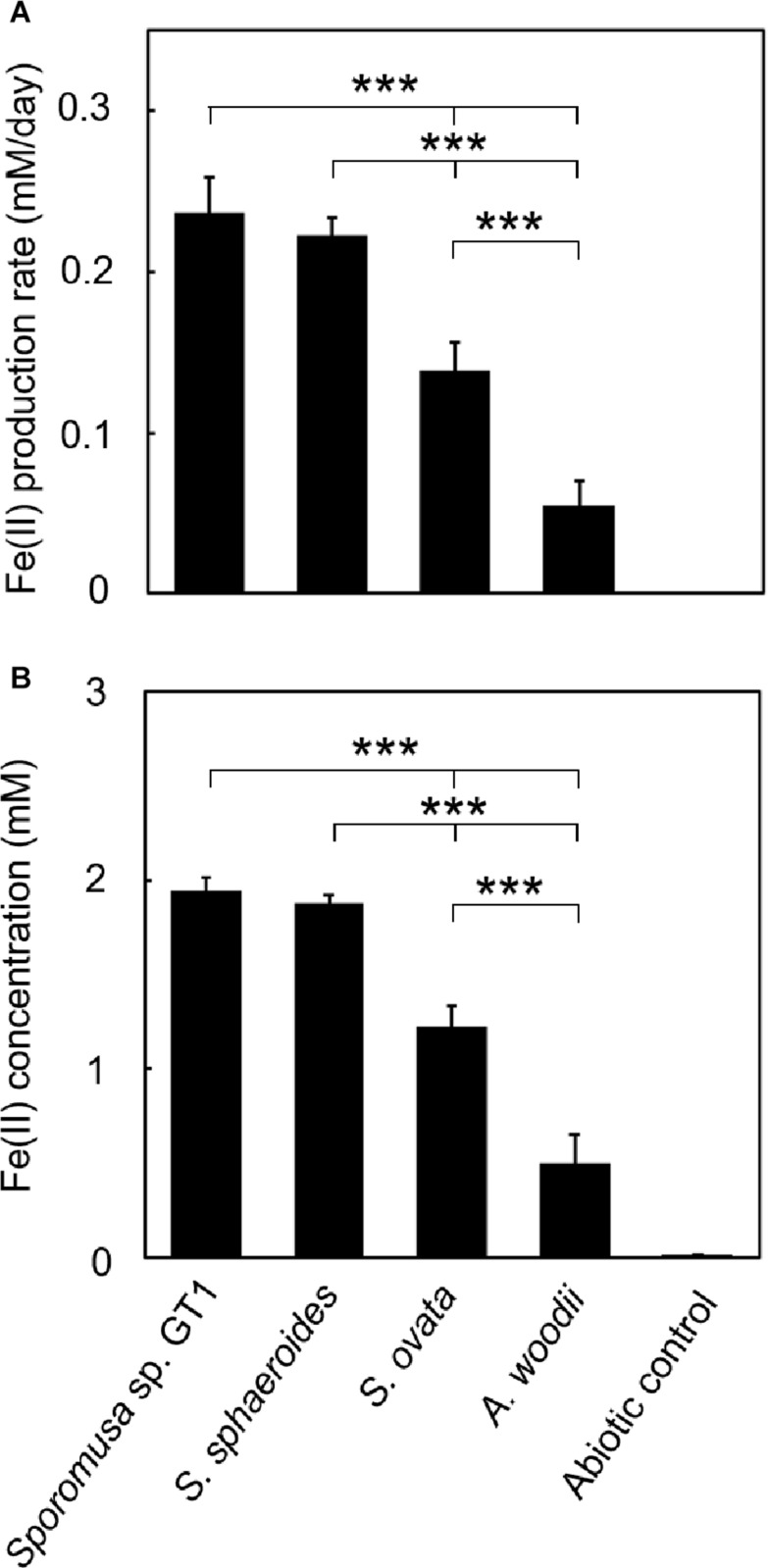
Difference in HFO reduction activity among homoacetogens under lithotrophic growth conditions. Homoacetogens were cultivated on H_2_ + CO_2_ (80:20) in the presence of HFO. **(A)** Fe(II) production rate in the exponential phase. **(B)** Fe(II) accumulation in the stationary phase. Data are presented as the means of three independent cultures, and error bars represent standard deviations. ***p < 0.001, determined by one-way analysis of variance with Tukey’s honest significance difference test.

To determine if HFO reduction was limited to organotrophic growth conditions, lithotrophic cultures were performed in which the growth substrate ethanol was replaced with H_2_ + CO_2_. All homoacetogens tested showed lithotrophic growth and stoichiometric acetogenesis according to Eq. 2 (Figure 4 and Supplementary Figure 3). HFO reduction was also observed under lithotrophic culture conditions. Similar to the organotrophic culture conditions, the highest HFO reduction activity was observed in the cultures of strain GT1 and *S. sphaeroides* (Figure 5). The Fe(II) production rates of strain GT1 and *S. sphaeroides* were 0.24 ± 0.02 and 0.23 ± 0.01 mM/day, respectively. The final Fe(II) concentration reached *ca*. 1.9 mM for both species. A limited portion of electrons (∼10%) from H_2_ was consumed for HFO reduction. Under lithotrophic growth conditions, 5–22% of the added Fe(III) was reduced to Fe(II) during the tested growth period (14 days). In addition, we observed reductive dissolution of HFO under lithotrophic growth conditions (Supplementary Figure 4).

### Reduction of Soluble Fe(III) and Crystalline Fe(III) Oxides

**FIGURE 6:**
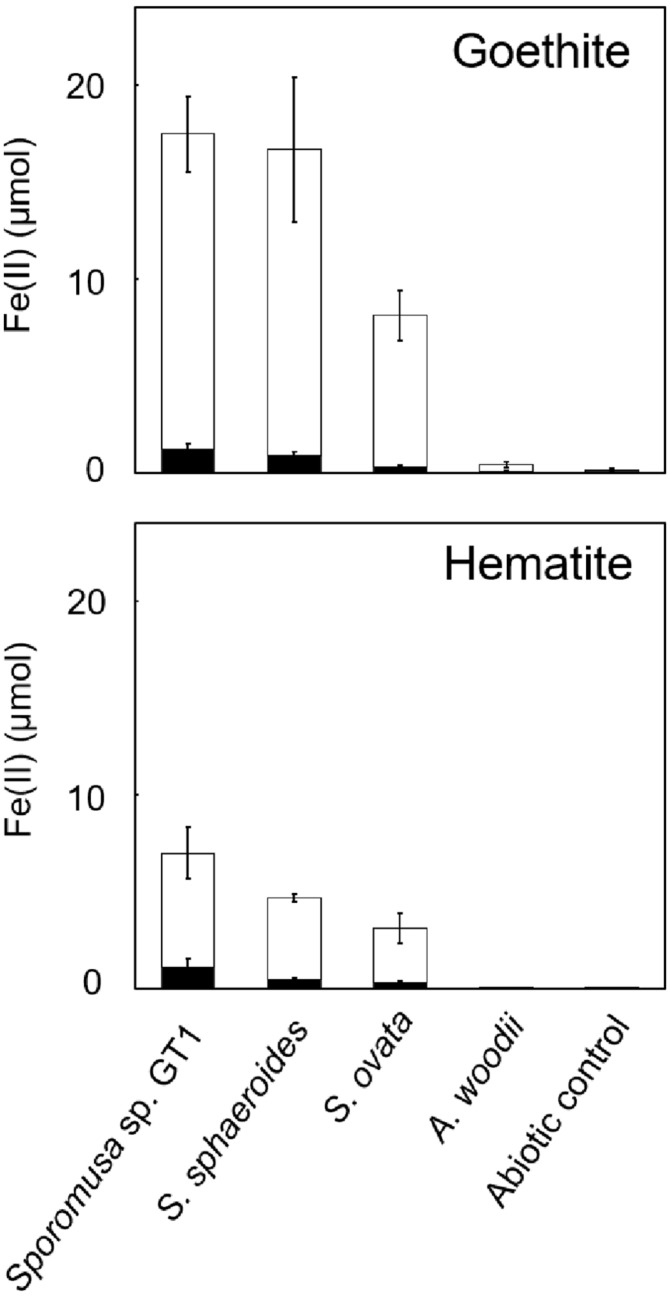
Difference in crystalline Fe(III) oxide reduction activity among homoacetogens under organotrophic growth conditions. Homoacetogens were cultivated on ethanol (20 mM) in the presence of goethite [α-FeO(OH), 9 mmol/L] or hematite (α-Fe_2_O_3_, 4.5 mmol/L). The white and black bars indicate Fe(II) contents in the supernatant and sediment, respectively. Data are presented as the means of three independent cultures, and error bars represent standard deviations.

As reported in previous studies, HFO-reducing bacteria can also reduce soluble Fe(III) and crystalline Fe(III) oxides. All homoacetogens showed Fe-NTA reduction activity similar to that with HFO (Supplementary Figure 5). Interestingly, although Fe(III) was provided in its soluble form, only 5–29% of soluble Fe(III) was reduced during cultivation, suggesting that the availability of soluble Fe(III) ion cannot be the rate-limiting step in Fe(III) reduction. We then tested two crystalline Fe(III) oxides (goethite and hematite). Although Fe(II) accumulation was dramatically decreased relative to that observed in cultures with HFO, strain GT1, *S. sphaeroides*, and *S. ovata* demonstrated the ability to reductively dissolve these crystalline Fe(III) oxides (Figure 6).

### Reductive Transformation of HFO

**FIGURE 7:**
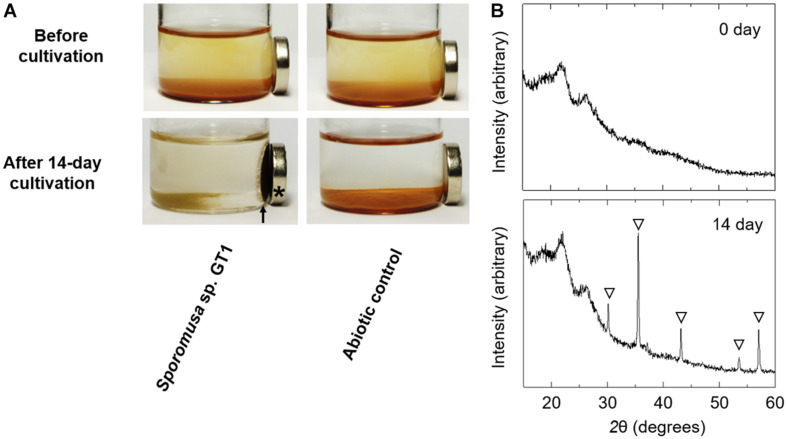
Transformation of HFO to magnetite by ethanol-grown culture of strain GT1. Strain GT1 was cultivated on ethanol (20 mM) in the presence of HFO under phosphate-limiting conditions. **(A)** Appearance culture bottles. Asterisk indicates a magnet. Arrow indicates magnet-attracted sediment. **(B)** Powder X-ray diffraction patterns of the sediments from the ethanol-grown culture of strain GT1. Open triangle, peaks from magnetite.

Powder XRD analysis revealed that the reduction product of HFO after both organotrophic (Supplementary Figure 6) and lithotrophic cultures (data not shown) of strain GT1 was X-ray silent, indicating that the Fe(II) form(s) produced were amorphous and/or soluble. When the stationary phase culture was further supplemented with 60 mM of ethanol (80 mM in total), HFO was almost completely dissolved by microbial reduction during prolonged cultivation (data not shown). This indicates that reduction of HFO by homoacetogens under the culture conditions tested induce reductive dissolution of HFO. As strain GT1 showed strong reduction activity, different culture conditions were evaluated using the strain. Cultivation under phosphate-limiting conditions showed almost the same growth, acetogenesis, and Fe(II) production as under phosphate-rich conditions, albeit at decreased rates (Supplementary Figure 7). Interestingly, throughout cultivation, most of the sediment containing Fe(III) was not dissolved and became ferrimagnetic after day 10 (Figure 7, for 14-day culture). XRD analysis revealed that the sediment contained magnetite. No other ferrimagnetic mineral was detected by XRD; thus, ferrimagnetism of the sediment likely accounted for the formed magnetite.

### Reduction Mechanism

**FIGURE 8:**
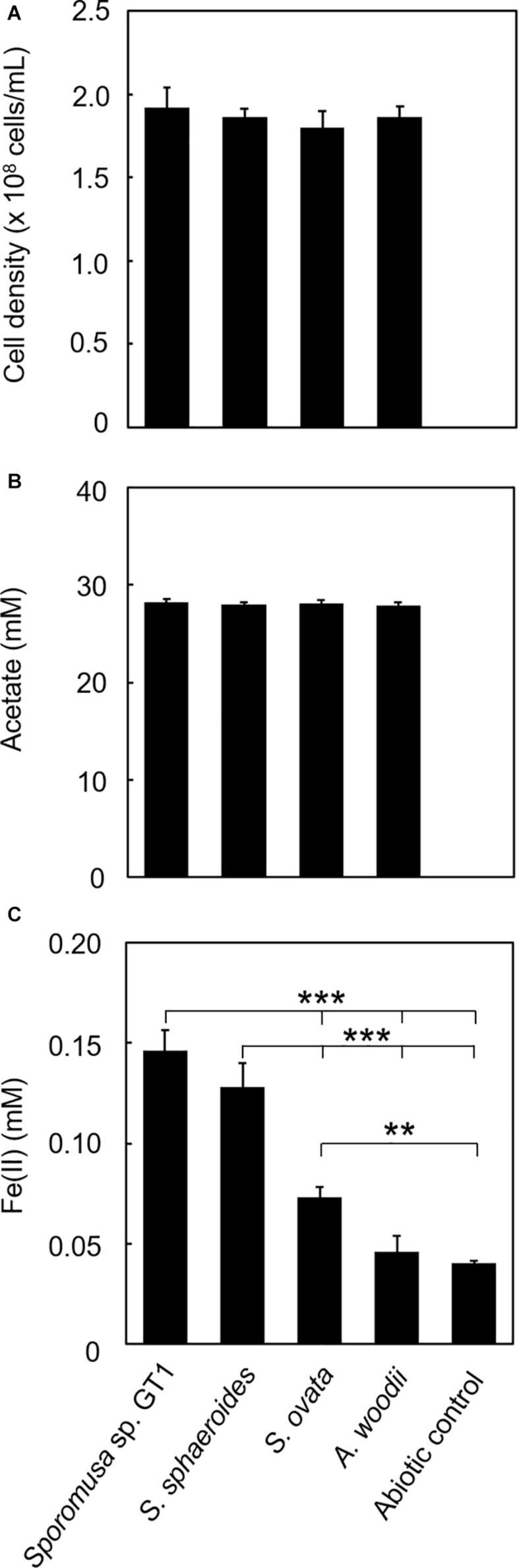
Effects of physical separation of HFO from the cells on reduction. Homoacetogens were cultivated with HFO on ethanol. Cultivation was conducted under the same conditions as described in section “Microorganisms and cultivations,” except that the culture medium volume was increased to 120 mL and HFO was placed inside the dialysis device. **(A)** Cell density, **(B)** acetate concentration, and **(C)** Fe(II) concentration were determined in the stationary phase. ***p < 0.001, **p < 0.01, determined by one-way analysis of variance with Tukey’s honest significance difference test.

For some members of IRBs, efficient Fe(III) reduction is possible by direct contact of the cell surface with Fe(III) oxide. To test if the homoacetogens require physical contact with Fe(III) oxide, cultivation was performed under conditions in which the cells and HFO were physically separated. For all homoacetogens, physical separation by the dialysis membrane still permitted Fe(III) reduction at *ca*. 0.06–0.3% of the total Fe(III) input, although the final Fe(II) production was dramatically decreased (Figure 8). This suggests that direct contact is the major pathway of Fe(III) reduction; however, a limited number of electrons can be transferred by a soluble compound(s) permeable to the dialysis membrane (<20 kDa).

To check if any extracellular compounds abiotically enhanced Fe(III) reduction, incubation using cell-free spent media from the cultures of each homoacetogen was performed. No Fe(III) reduction was observed compared to the control incubated without spent medium even after 30 days of incubation (Supplementary Figure 8). This indicates that microbial metabolism was involved in Fe(III) reduction, eliminating the possibility of chemical reduction mediated by soluble compounds excreted from the cells.

Flavins are reportedly candidate molecules for indirect Fe(III) reduction; therefore, we performed cultivations in the presence of riboflavin or FAD. Neither flavin (5 μM) enhanced Fe(III) reduction under organotrophic (Supplementary Figure 9) or lithotrophic conditions (Supplementary Figure 10). This result indicated that the Fe(III) reduction observed in this study was independent of the flavin-based EET mechanism reported previously (Light et al., 2018; Li et al., 2020).

### Comparative Genomic Analysis

To examine the mechanism underlying Fe(III) reduction by homoacetogens, comparative genomic analysis was conducted. Since genomic information for strain GT1 was not publicly available, we first determined the draft genome sequence of this strain. The draft genome harbored 119 contigs with a total length of 4,771,584 base pairs, G + C content of 47.3%, and sequencing coverage of 159-fold. An *N*_50_ value of 152,441 base pairs was obtained. Among the 4,435 total predicted genes, 4,332 were protein-coding genes, 99 were tRNA genes, and 3 were rRNA genes.

Along with the genome of strain GT1, the genomes of the other three homoacetogens retrieved from the NCBI database were analyzed. Typical IRBs that can perform direct electron transfer employ MHCs to establish the electron path connecting the inner cell membrane to the outer cell surface. For example, *Thermincola potens* JR, a well-known iron-reducing bacterium in the class *Clostridia*, possesses 35 MHCs with common heme-binding domain (CxxCH), enabling direct electron transfer to Fe(III) oxides. Thus, the presence of MHCs with this domain was investigated in the genome sequence of the four homoacetogens. Table 1 summarizes the representative homoacetogens reported to conduct insoluble Fe(III) reduction and/or Fe(0) oxidation, with their relevant cellular characteristics. MHCs with CxxCH domain were detected in the genomes of three homoacetogens tested in this study: four in strain GT1, four in *S. sphaeroides*, and eight in *S. ovata*. However, neither MHC possessed over five hemes. These MHCs are expected to be membrane-bound (Supplementary Table 1). *Acetobacterium woodii* contained no MHC even though HFO reduction was observed; this is like *Clostridium* sp. FGH, which reduces HFO (Shah, 2013). We then performed further analyses of variant heme-binding domains (CxxxCH, CxxxxCH, CxxCK, and A/FxxCH). Although this analysis detected redox proteins with multiple heme-binding domains (i.e., cytochromes and iron-sulfur proteins), no MHCs with six or more heme-binding domains were observed (Supplementary Table 2). The results also indicated that the capacity for insoluble iron reduction by the homoacetogens was not directly correlated with the number of MHCs in the genomes.

**TABLE 1:** Summary of predicted multiheme *c*-type cytochromes in homoacetogens.

Acetogens	Outer membrane	Oxidation of Fe^0^	Reduction of Fe(III) oxide	Predicted MHCs
*Sporomusa* sp. GT1	N.R.	Yes^*c*^	Yes^*d*^	4^*d*^
*Sporomusa sphaeroides* (DSM2875)	Yes^*a*^	Yes^*c*^	Yes^*d*^	4^*d*^
*Sporomusa ovata* (DSM2662)	Yes^*a*^	No^*c*^	Yes^*d*^	8^*d*^
*Acetobacterium woodii* (DSM1030)	N.R.	No^*c*^	Yes^*d*^	0^*d*^
*Clostridium* sp. FGH	N.R.	N.R.	Yes^*e*^	0^*e*^
*Thermincola potens* JR	No^*b*^	N.R.	Yes^*f*^	35^*b*^

*^a^Möller et al., 1984^b^Carlson et al., 2012^c^Kato et al., 2015^d^This study ^e^Shah, 2013^f^Wrighton et al., 2011 N.R., not reported. Heme-binding domain (cytochrome motif) was searched over genomes of homoacetogens used in this study. Clostridium sp. FGH and Thermincola potens JR were also analyzed for comparison.*

We then analyzed representative genes required for dissimilatory iron reduction *via* MHC-dependent electron transport in the genomes of the homoacetogens. Although the four homoacetogens possess *pilA* homologs, other membrane-associated EET proteins, such as *mtrABC* and *omsS*, were completely or partially absent from the genomes (Supplementary Table 3). In addition, the riboflavin biosynthesis pathway was conserved in the four homoacetogens (Supplementary Table 4); however, no clear evidence of the presence of gene sets necessary for flavin-based EET in Gram-positive bacteria was found in the strain GT1, *S. sphaeroides*, or *A. woodii* due to the absence of *ndh2*, which plays a central role in flavin-based EET (Supplementary Table 5). These results suggest the likelihood that Fe(III) reduction by homoacetogens is mediated by neither membrane-bound MHCs reported in well-known Gram-negative DIRBs nor flavin-based EET in Gram-positive bacteria.

## Discussion

This study demonstrated that three *Sporomusa* spp. and one *Acetobacterium* bacterium can reduce insoluble Fe(III) (i.e., HFO) under both organotrophic and lithotrophic conditions. Previous studies indicated that fermentative bacteria perform Fe(III) reduction by discarding excess reducing equivalents as a form of enhanced fermentation (Lovley et al., 2004; Dalla Vecchia et al., 2014). In addition, List et al. (2019) suggested that Fe(III) reduction can function as buffer reaction against pH changes in the growth environment, resulting in acidogenesis by fermentation to persist for longer. However, our results showed that the presence of Fe(III) does not critically affect acetogenesis and growth, indicating that the Fe(III) reduction observed in this study is a side reaction for electron transfer not directly linked to energy conservation, as reported in previous studies (Shah, 2013; Dalla Vecchia et al., 2014). In addition, our culture medium did not exhibit large pH shifts (maximum of 0.6 units decrease from 7.4) in both HFO-amended culture and control culture because of the strong pH buffering action (see “Microorganisms and cultivations”). Thus, Fe(III) reduction during the growth is not considered as a beneficial metabolism pathway for homoacetogens, at least under our experimental conditions.

Separation of HFO with dialysis membrane still allowed HFO reduction by all homoacetogens tested, although the amount of Fe(II) produced was decreased. This indicates that the Fe(III) reduction mechanisms of these homoacetogens involve in the function of soluble compounds as electron mediators, and proximity between the Fe oxide surface and cell substantially increases the reduction efficiency. Furthermore, the spent medium of the cultures of homoacetogens did not induce abiotic HFO reduction, indicating that the functions of soluble electron mediators depend on metabolism rather than being chemically active. This observation follows those of a previous study using *Clostridium* sp. FGH (Shah, 2013). In addition, *Desulfotomaculum reducens* strain MI-1, a bacterium in the order *Clostridiales*, reduces Fe(III) *via* a process involving a soluble electron carrier, most likely riboflavin (Dalla Vecchia et al., 2014). Several studies reported that *Shewanella* strains can excrete flavin mononucleotide (0.45 kDa) and riboflavin which are soluble molecules that function as electron shuttles for Fe(III) oxide reduction (Marsili et al., 2008; von Canstein et al., 2008). Recently, the involvement of polyphosphate, a ubiquitous inorganic polyanion, was suggested for Fe chelation (Beaufay et al., 2020). Therefore, these molecules and/or other unidentified molecules are involved in Fe(III) reduction by the homoacetogens observed in this study.

Genomic analysis revealed that the four homoacetogens capable of direct HFO reduction use different mechanisms from other well-known IRBs, such as *Geobacter*, *Shewanella*, and *Thermincola* spp., which possess dozens of membrane-bound MHCs with over six hemes per cytochrome. Furthermore, flavin-based EET mechanisms are assumed to be uninvolved in the Fe(III) reduction observed in this study (Supplementary Figures 9, 10 and Supplementary Table 5). The primary mechanism of reduction by homoacetogens is likely mediated by enzymes on the outer cell surface and that indirectly transfer electrons to HFO based on the following observations: (1) HFO reduction by the homoacetogens tested was evident when the cells had direct access to the HFO surface, and (2) homoacetogens that showed strong Fe(III)-reduction ability possess membrane proteins with MHCs. The secondary Fe(III)-reduction mechanism would be mediated by a soluble molecule(s) other than flavins (Figure 8). Interestingly, the ability for HFO reduction is correlated with the ability for Fe(0) oxidation, particularly in strain GT1 and *S. sphaeroides* (Kato et al., 2015; Figures 3, 5). Thus, an unknown electron transfer mechanism mediating direct electron transfer may be shared with iron reduction and oxidation in these species. Further comparative genomics studies combined with transcriptome and proteome analysis are needed to reveal the detailed mechanisms of iron reduction by these homoacetogens and other mesophilic homoacetogens.

Reduction products generated by homoacetogens in this study were soluble Fe(II) and an amorphous form of Fe(II) in phosphate-rich conditions. Dissolution and secondary mineralization of the reduction products are strongly influenced by the carbonate, phosphate, and organic matter concentrations, as well as by pH and temperature (Couling and Mann, 1985; Fredrickson et al., 1998; Kukkadapu et al., 2004). Magnetite formation under phosphate-limiting conditions suggests that phosphate inhibited secondary mineralization of Fe(II) to form magnetite *via* a similar mineralogical process reported previously (Couling and Mann, 1985). Our results also suggest the potential involvement of *Sporomusa* species in the magnetite formation, which may help to form the environments where direct interspecies electron transfer occurs in anaerobic soil and sedimentary environments (Kato et al., 2012a, b; Kato, 2015).

It is noteworthy that all homoacetogens tested in this study, except for *A. woodii*, reduced crystalline Fe(III) oxides (goethite and hematite) (Figure 6), because only limited members of IRBs, in particular, bacteria belonging to *Geobacter* and *Shewanella*, can directly reduce crystalline Fe(III) (Roden and Urrutia, 2002; Behrends and van Cappellen, 2007; Yan et al., 2008; Bose et al., 2009; Aneksampant et al., 2020; Hu et al., 2020). Results in this study broaden our knowledge on the role of mesophilic homoacetogens in the reduction and dissolution of crystalline Fe(III) oxides in nature.

Our results demonstrate that *Sporomusa* spp. can induce Fe(III) oxide dissolution and transformation depending on the surrounding physical and chemical conditions, supporting their important role in the iron cycle and other elemental cycles. Considering that homoacetogens dominate in various anoxic environments where Fe(III) oxides accumulate, homoacetogens would benefit from Fe(III) reduction and/or relevant microbial interactions. A potential benefit of homoacetogens would be the active dissolution of insoluble Fe(III), including crystalline Fe(III) oxides, to obtain iron for metabolism. Metatranscriptomic approaches with stable isotope labeling experiments (Friedrich, 2006; Hori et al., 2007) can reveal the actual relationships between iron minerals and homoacetogens in nature.

## Data Availability Statement

The raw data sets for the Illumina HiSeq 2500 run are available in GenBank under accession number DRA010698. Assembled reads are available in GenBank Sequence Read Archive under accession numbers BMBX01000001–BMBX01000119.

## Author Contributions

KI and SK designed the experiments, interpreted the data, and contributed to the editing of the manuscript. KI performed the experiments and wrote the initial draft of the manuscript. Both authors contributed to the article and approved the submitted version.

## Conflict of Interest

The authors declare that the research was conducted in the absence of any commercial or financial relationships that could be construed as a potential conflict of interest.

## Footnotes

1 https://prosite.expasy.org/scanprosite/

2 https://supfam.mrc-lmb.cam.ac.uk/SUPERFAMILY/
